# Bridging Aging and Intellectual Disabilities Research: From Past Lessons to Future Directions

**DOI:** 10.1093/geroni/igaf122.977

**Published:** 2025-12-31

**Authors:** Tamar Heller

**Affiliations:** University of Illinois Chicago, Chicago, Illinois, United States

## Abstract

This presentation provides a historical overview of efforts to bridge aging and intellectual disability, highlighting key moments in more than 40 years of research. It discusses trends in aging among persons with intellectual disabilities and what increased longevity has meant for families and communities. Research and policy initiatives that have forwarded bridging over time are identified. Examples of evidence-based knowledge and practice interventions that demonstrate realized outcomes of bridged aging and disability research such as future planning and health promotion are provided. Recommendations for the future focus on addressing knowledge gaps and clarifying why bridged research is needed in areas such as family support, health care, and participation.

